# Jumping performance and muscle–tendon characteristics of Maasai men of East Africa

**DOI:** 10.1038/s41598-025-01694-9

**Published:** 2025-05-30

**Authors:** J. Bojsen-Møller, O. R. Seynnes, S. P. Magnusson, H. Hernæs, A. S. Refsdal, J. Sironga, V. P. Maro, K. Ramaiya, D. L. Christensen, P. Aagaard

**Affiliations:** 1 Norwegian School of Sport Sciences, Oslo, Norway; 2 Department of Sports Science and Clinical Biomechanics, University of Southern Denmark, Odense, Denmark; 3 Copenhagen University Hospital (Bispebjerg), Copenhagen, Denmark; 4 Monduli District Hospital, Monduli, Tanzania; 5 Kilimanjaro Christian Medical University College, Moshi, Tanzania; 6 Shree Hindu Mandal Hospital, Dar es Salaam, Tanzania; 7 University of Copenhagen, Copenhagen, Denmark

**Keywords:** Counter-movement jump, Repetitive jumping, Tendon stiffness, Physiology, Anatomy

## Abstract

The male Maasai of East Africa perform a ritual of repetitive jumping ceremonies and are known anecdotally for outstanding jumping performance. The aim of the present study was to assess vertical jumping ability and anatomical/biomechanical characteristics of Maasai jumpers. Twenty-two Maasai men performed maximal vertical countermovement jumps (CMJ), and repetitive jumps (RJ) on an instrumented force plate. Twelve age-matched Norwegian men served as controls (CON). Anthropometrics, kinematic/kinetic and electromyographic data were recorded during jumping. Resting and dynamic plantarflexor muscle architecture and Achilles tendon stiffness were measured using ultrasonography. Maximal jump height (CMJ and RJ) was similar between groups, however the Maasai jumped faster, with less vertical displacement, and greater ground-reaction peak forces and power in CMJ. In contrast, greater vertical displacement in RJ was seen for the Maasai compared to CON. The Maasai demonstrated longer relative leg length, and tendon structures, but lower fascicle pennation angles, shorter fascicle lengths and more compliant tendons. Although maximal jump height was similar, jump kinetics and kinematics differed between groups, which may relate to jumping tradition or to anthropometrical and/or muscle–tendon morphological/mechanical characteristics. Overall, the observed muscle–tendon characteristics of the Maasai may favor economy of movement during walking and perhaps during RJ.

## Introduction

At the population level, the Maasai people of Tanzania and Kenya are physically active, yet demonstrate low to moderate aerobic fitness levels^1^, which likely is related to the relatively low intensity of daily physical activity, demonstrated by the limited time spent (~ 13% of total physical activity energy expenditure) at moderate-to-high intensity physical activity (≥ 3 metabolic equivalents)^2^. Walking is the main mode of activity during herding of domesticated animals^3,4^. The Maasai are also known for their ritual jumping-dance activities mostly, but not only, performed by young unmarried men, so-called *Morans* (warriors in Maasai language)^5^. This culturally important activity is a crucial part of the identity of the Maasai, and has a long tradition that likely originates from when the Maasai people emerged from migrants of present-day South Sudanese and Ethiopian pastoralists about 500 years ago^6^. Anthropological accounts have described jumping-dance activities that last several days^7^, and a recent study from our group has reported jumping dance activity (on the same Maasai participants as those of the current study) showing that they amount to 28% of total daily physical activity when performed^8^. With respect to biomechanics, one recent study has examined jumping-dance rituals based on video material^9^, but there are no detailed biomechanical studies of Maasai jumping. It seems that the jumping efforts of the Maasai are mainly repetitive in nature, and despite anecdotal reports of the extreme maximal jumping capability (jump height) of the Maasai^10^, no scientific documentation could be found to describe the jumping performance of the Maasai.

Over the past century, a number of studies have reported general anthropometric characteristics of the Maasai, such as height, weight, and foot and shank morphometry^2,11–13^, whereas more detailed data of anthropometry, including lower limb muscle–tendon biomechanical and anatomical/anthropometric variables of importance for human jumping performance appear to be lacking. Therefore, the present study aimed to (1) examine the vertical jumping ability of male Maasai jumpers during standardized maximal and repetitive jumping efforts, and (2) to assess biomechanical and anatomical lower limb characteristics in this population. Based on previous reports on jumping capability^9,10^, we hypothesized that the Maasai would demonstrate a superior ability for maximal vertical jumping (jump height) both in maximal single jumping efforts, and in repetitive jumping compared to a Norwegian group of control participants. Furthermore, based on previous studies that report on the relation between anthropometry/muscle–tendon biomechanics and jumping performance^14–19^ we hypothesized that the Maasai would have distinct anthropometric characteristics (segment dimensions) and muscle-architectural and tendon biomechanical properties (mechanical properties of force bearing tissues) of the lower limb that would facilitate vertical jump performance.

## Results

Age was similar for the Maasai and control (CON) participants, while height, weight and body mass index (BMI) were greater in CON (*P* < 0.002) (Table 1).

**Table 1:** Anthropometry.

	Maasai (n = 22)	Controls (n = 12)	95% C.I.	*P*
Age (yrs)	26.6 ± 6.0	25.8 ± 4.9	− 3.5, 4.9	0.746
Height (cm)	170.1 ± 7.0	177.6 ± 4.5	− 12.1, − 2.9	0.002*
Weight (kg)	55.8 ± 6.7	69.3 ± 4.4	− 17.9, − 9.1	< 0.001*
BMI (kg/m^2^)	19.3 ± 1.7	22.0 ± 1.9	− 4.0, − 1.4	< 0.001*
Leg length (cm)	84.1 ± 5.2	83.9 ± 4.3	− 3.4, 3.9	0.887
Thigh length (cm)	41.3 ± 2.4	41.6 ± 2.0	− 2.0, 1.4	0.700
Shank length (cm)	42.8 ± 3.2	42.2 ± 2.5	− 1.9, 1.3	0.596
Foot length (mm)	260.4 ± 14.5	261.3 ± 12.5	− 11.2, 9.4	0.867
AT M arm (mm)	47.3 ± 3.3	50.7 ± 3.2	− 6.5, − 1.2	0.008*
Leg length/height (%)	49.4 ± 1.7	47.2 ± 1.4	1.0, 3.4	< 0.001*
Shank length/height (%)	25.2 ± 1.1	23.8 ± 0.8	0.7, 2.1	< 0.001*
Foot length/height (%)	15. 3 ± 0.6	14.7 ± 0.5	0.2, 1.0	0.005*
AT M arm/Shank length (%)	11.1 ± 0.8	12.0 ± 0.9	− 1.5, − 0.3	0.005*

Group mean ± SD. 95% confidence interval (C.I) for differences between means.

ATM, arm achilles tendon moment arm; BMI, body mass index.

*Denotes significant group difference.

### Anatomical lower limb characteristics

Leg, thigh and shank leg length were similar between groups, but total leg length/height and shank length/height was ~ 5 and 6% greater in the Maasai (*P* < 0.001). Foot length was similar between groups, however when normalized to body height it was ~ 4% greater in Maasai compared to CON (*P* = 0.005). The Achilles tendon moment arm was ~ 7% shorter in the Maasai both in absolute values and when normalized to shank length (*P* = 0.008, *P* = 0.005 respectively) (Table 1).

Resting ankle joint angle (relative to in situ position (0°)) when lying in prone position was 7° less plantarflexed (PF) in the Maasai (27 ± 5° PF) compared to CON (34 ± 2° PF) (*P* < 0.001, (− 10.1, − 3.9)).

### Proximo-distal muscle distribution

**Fig. 1:**
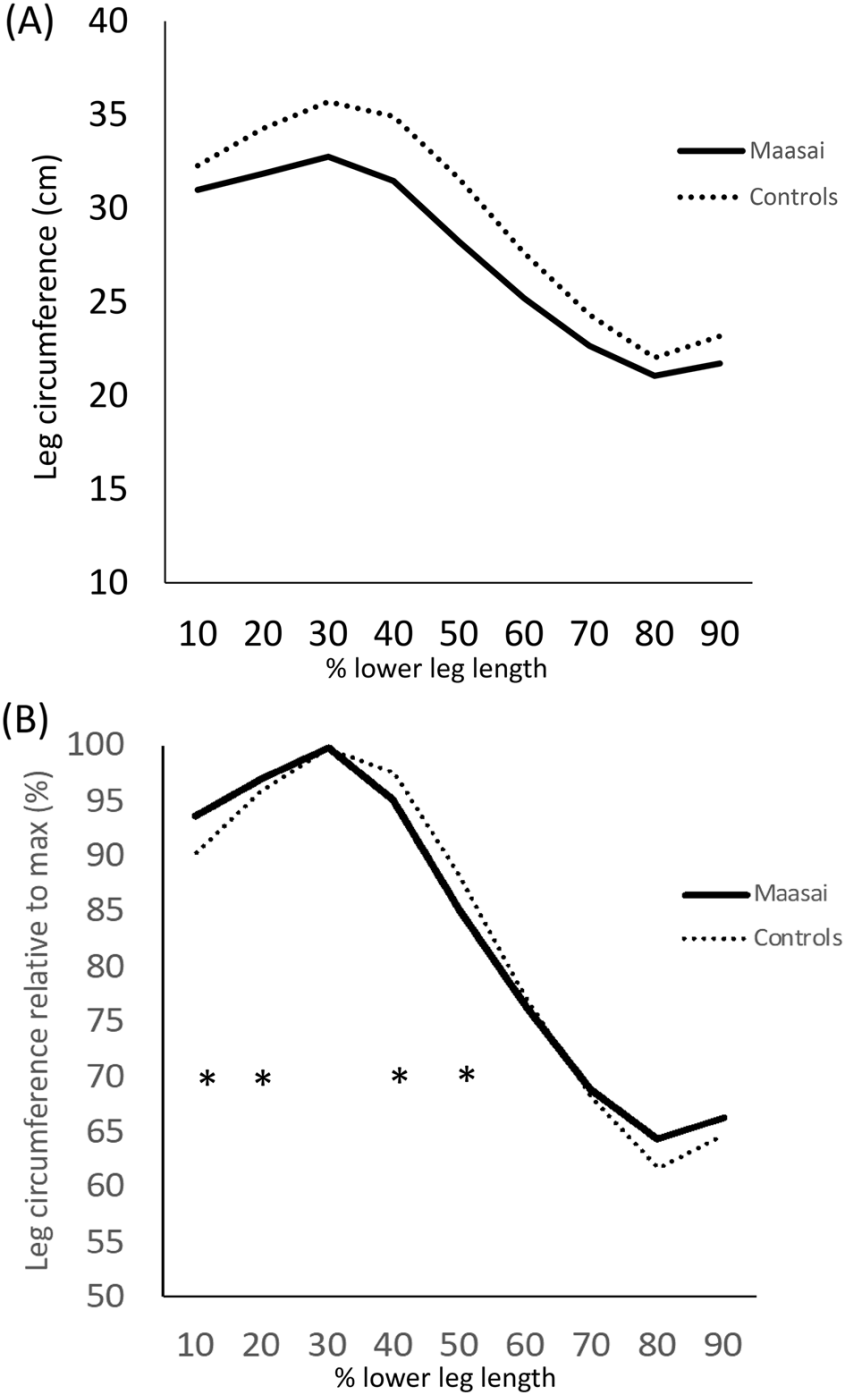
Shank circumference measured at 10% increments from the knee joint to the ankle. (**A**) Absolute leg circumference (cm). (**B**) Relative (to greatest individual value) circumference (%). * Maasai vs. CON (*P* < 0.01).

The absolute calf volume (cumulated circumference) was ~ 8% less in the Maasai compared to CON; 246 ± 12 vs, 265 ± 9 cm, (*P* = 0.001, (− 27.2, − 10.8)). Calf volume was more proximally distributed in Maasai participants, manifested by a larger relative shank circumference in Maasai versus CON when assessed at the 10 and 20% marks (distance to the knee joint line) (94 ± 3 and 97 ± 2 vs. 90 ± 3 and 96 ± 1% (*P* = 0.03, (2.2, 5.8)), while conversely relative shank circumference was less in the Maasai than CON when assessed distal (40 and 50% marks) to the knee joint (95 ± 2 and 85 ± 3 vs. 98 ± 2 and 88 ± 4% (P = 0.03, 4.8, 9.1)) (Fig. 1).

### Resting muscle architecture

Maasai participants demonstrated less pennate (9%, *P* = 0.048) and shorter GM fascicle lengths (10%, *P* = 0.047) compared to CON. Similarly, GM muscle thickness was 17% lower in Maasai compared to CON (*P* = 0.001). No differences in VL muscle architecture were observed between the groups (Table 2).

**Table 2:** Resting muscle architecture.

	Maasai (n = 19)	Controls (n = 12)	95% C.I.	*P*
GM fascicle length (mm)	48.3 ± 5.8	53.4 ± 7.9	− 10.1, − 0.1	0.047*
GM pennation angle (°)	19.5 ± 2.4	21.4 ± 2.8	− 3.8, 0.0	0.048*
GM thickness (mm)	15.0 ± 2.2	18.4 ± 1.7	− 4.9, − 1.9	< 0.001*
VL fascicle length (mm)	78.7 ± 7.9	81.7 ± 15.7	− 11.7, 5.7	0.485
VL pennation angle (°)	15.3 ± 1.9	16.0 ± 3.4	− 2.6, 1.2	0.497
VL thickness (mm)	21.2 ± 2.4	21.6 ± 3.9	− 2.7, 1.9	0.678

Group mean ± SD. 95% confidence interval (C.I.) for differences between means.

GM, medial gastrocnemius; VL, vastus lateralis.

*Denotes significant group difference.

### Achilles tendon properties

**Fig. 2:**
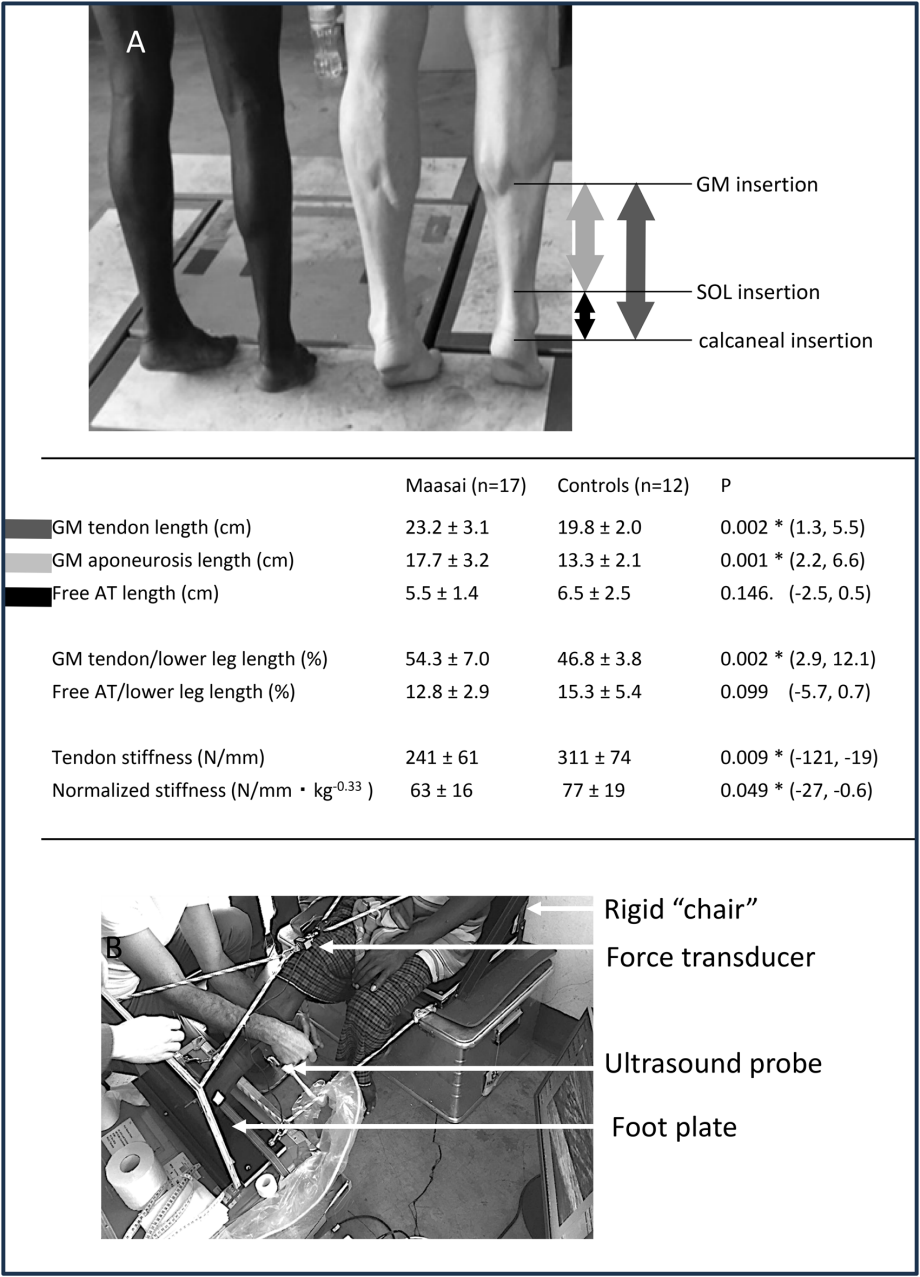
(**A**) Achilles tendon (AT) properties. Group means ± SD. The dark grey arrow denotes the GM tendon i.e., tendinous tissue length from the most distal point of GM insertion on the aponeurosis to the calcaneal insertion. The black arrow denotes the free AT length i.e., the distance from the most distal insertion of SOL on the tendon to the calcaneal insertion, while the light grey arrow denotes the GM aponeurosis i.e., the difference between the two structures (Note that the current image was included for illustrational purposes and shows a Maasai study participant alongside one of the authors. It was not possible to depict a Maasai and a CON participant in one photo, since CON participants were examined in Norway). (**B**) The portable isometric plantarflexion device designed for measurement of tendon stiffness. The present image shows the participant resting (flexed knee) between plantarflexor attempts. During measurement the knee joint was extended and firm fixation of the limb was attained.

Free Achilles tendon length was similar in the two groups, whereas GM tendon and GM aponeurosis lengths were greater in the Maasai (~ 17 & 33%, respectively; *P* = 0.002 and < 0.001) (Fig. 2). Likewise, when normalized relative to shank length, GM tendon and GM aponeurosis lengths were longer in Maasai vs. CON (P < 0.001), while no group difference was observed for normalized length of the free Achilles tendon.

The absolute and the normalized (to bodyweight^19^, (kg^0.33^)) stiffness of the GM tendon was 23% and 18% lower in Maasai compared to CON (*P* = 0.001/*P* = 0.04) respectively (Fig. 2).

### CMJ performance

Maximal vertical jump height was similar between Maasai and CON (Table 3), however when expressed relative to body mass, the Maasai participants jumped ~ 20% higher than CON (80 ± 9 vs. 67 ± 9 cm/kg, *P* < 0.001). A shorter duration (~ 42%) of the CMJ take-off phase was observed in the Maasai (*P* < 0.001). The fast take-off was accomplished by a reduced displacement of body center of mass (BCM) during the flexion (BCM descending downwards) and extension (BCM ascending upwards) take-off phases, (~ 21 & 29%, respectively) BCM displacement (*P* < 0.001), and a ~ 23 & 24% greater (flexion and extension phase, respectively) peak vertical take-off force (peak F_z_ normalized to body mass) (P < 0.01) in Maasai participants. Likewise, the mean force in the extension phase was similarly greater in the Maasai (~ 17%, *p* = 0.012) (Table 3).

**Table 3:** Maximal countermovement jumping.

	Maasai (n = 15)	Controls (n = 12)	95% C.I.	*P*
Kinematics				
Maximal jump height (cm)	46.5 ± 4.3	46.5 ± 6.2	− 4.2, 4.2	0.987
Duration FLX_dec_ (ms)	114 ± 29	172 ± 22	− 79, − 39	< 0.001*
Duration EXT (ms)	213 ± 38	286 ± 43	− 105, − 41	0.001*
ΔBCM FLX (cm)	24.6 ± 4.2	34.8 ± 6.8	− 15.5, − 4.9	0.001*
ΔBCM EXT (cm)	36.1 ± 5.1	45.9 ± 8.1	− 14.4, − 4.3	0.001*
Hip joint ROM (°)	81.1 ± 13.3	105.8 ± 20.9	− 38.3, − 11.1	< 0.001*
Knee joint ROM (°)	80.2 ± 10.8	87.2 ± 13.2	− 16.5, 2.5	0.112
Ankle joint ROM (°)	26.1 ± 5.0	25.2 ± 6.2	− 3.5, 5.3	0.657
Kinetics				
Peak F_Z_ force FLX (N/kg)	29.2 ± 5.2	23.6 ± 1.4	2.4, 8.8	0.011*
Peak F_Z_ force EXT (N/kg)	29.7 ± 5.3	24.0 ± 1.4	2.4, 8.7	0.010*
Mean F_Z_ force EXT (N/kg)	22.3 ± 2.9	19.1 ± 1.5	1.3, 5.1	0.012*
Power, work				
Peak power EXT (W/kg)	55.2 ± 8.3	49.2 ± 5.7	0.2, 11.8	0.050*
F_Z_ @ peak power (N/kg)	23.1 ± 3.3	19.7 ± 1.5	1.3, 5.5	0.003*
V_BCM_ @ peak power (m/s)	2.39 ± 0.14	2.49 ± 2.18	− 1.3, 1.1	0.119
Mean power EXT (W/kg)	32.9 ± 5.6	27.8 ± 3.3	1.3, 8.9	0.001*
Work FLX (J/kg)	6.68 ± 0.65	7.29 ± 1.19	− 1.4, 0.1	0.001*
Work EXT (J/kg)	2.41 ± 5.3	3.42 ± 0.69	− 4.2, 2.2	0.008*
Ratio work EXT/work FLX	2.90 ± 0.37	2.35 ± 0.22	0.3, 0.8	< 0.001*
*RFD, stiffness, efficiency*				
RFD (N/s kg)	219.9 ± 199.2	88.6 ± 28.2	11.5, 251.1	0.001*
LLS (N/m kg)	245.6 ± 135.1	96.0 ± 25.6	67.8, 231.4	0.001*
RSI FLX_dec_ + EXT (m/s)	1.48 ± 0.36	1.01 ± 0.17	0.2, 0.7	< 0.001*
JH/work (cm/(J/kg))	5.10 ± 0.50	4.51 ± 0.45	0.2, 1.0	0.004*

Group mean ± SD. 95% confidence interval (C.I.) for differences between means.

FLX_dec_, deceleration during flexion phase; FLX, flexion phase (BCM moving downwards); EXT, extension phase (BCM moving upwards); ΔBCM, displacement body center of mass. Joint excursion is presented as maximal flexion (hip and knee joint) and maximal dorsiflexion (ankle joint) ROM during the push off phase relative to in situ joint position. F_z_ force, vertical ground reaction force; V_BCM_, BCM velocity; RFD, rate of Fz development measured from onset of FLX_dec_ to + 50 ms; LLS, lower limb stiffness calculated as ΔF_z_ /ΔBCM; RSI FLX_dec_ + EXT, Reactive Strength Index calculated as jumpheight (m)/duration of the FLX_dec_ + EXT phases; JH/Work, maximal vertical jump height normalized to work produced in the extension phase (vertical jump efficiency).

*Denotes significant group difference.

**Fig. 3:**
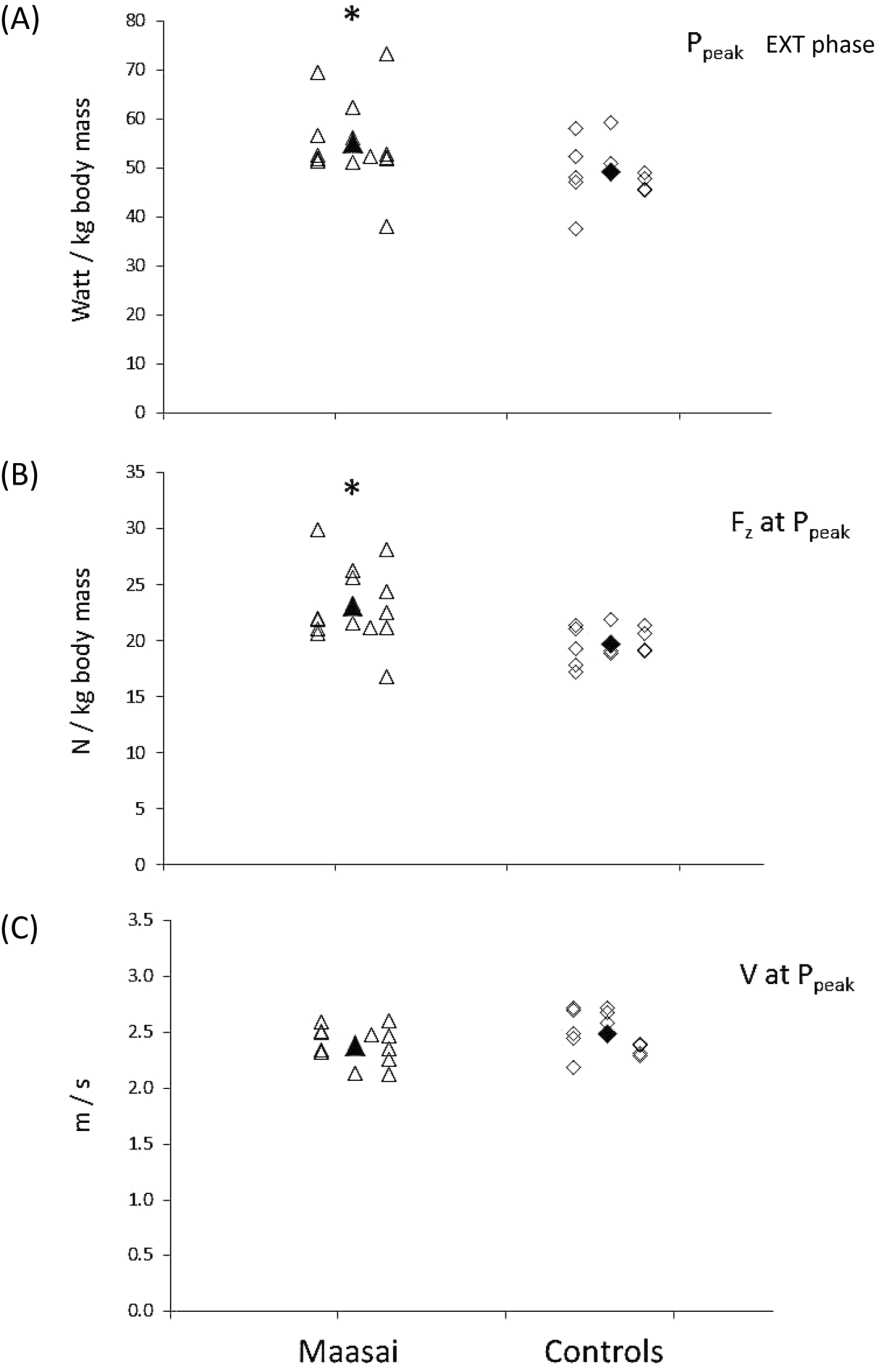
Countermovement jumping performance in Maasai (triangles) and CON (diamonds). Mean data of the groups are depicted by solid symbols. (**A**) Peak power (P_peak_) recorded in the extension phase of the take-off, (**B**) ground reaction force (F_z_ ) at P_peak_, (**C**) BCM velocity at P_peak_. * Maasai vs. CON (*P* < 0.01).

**Fig. 4:**
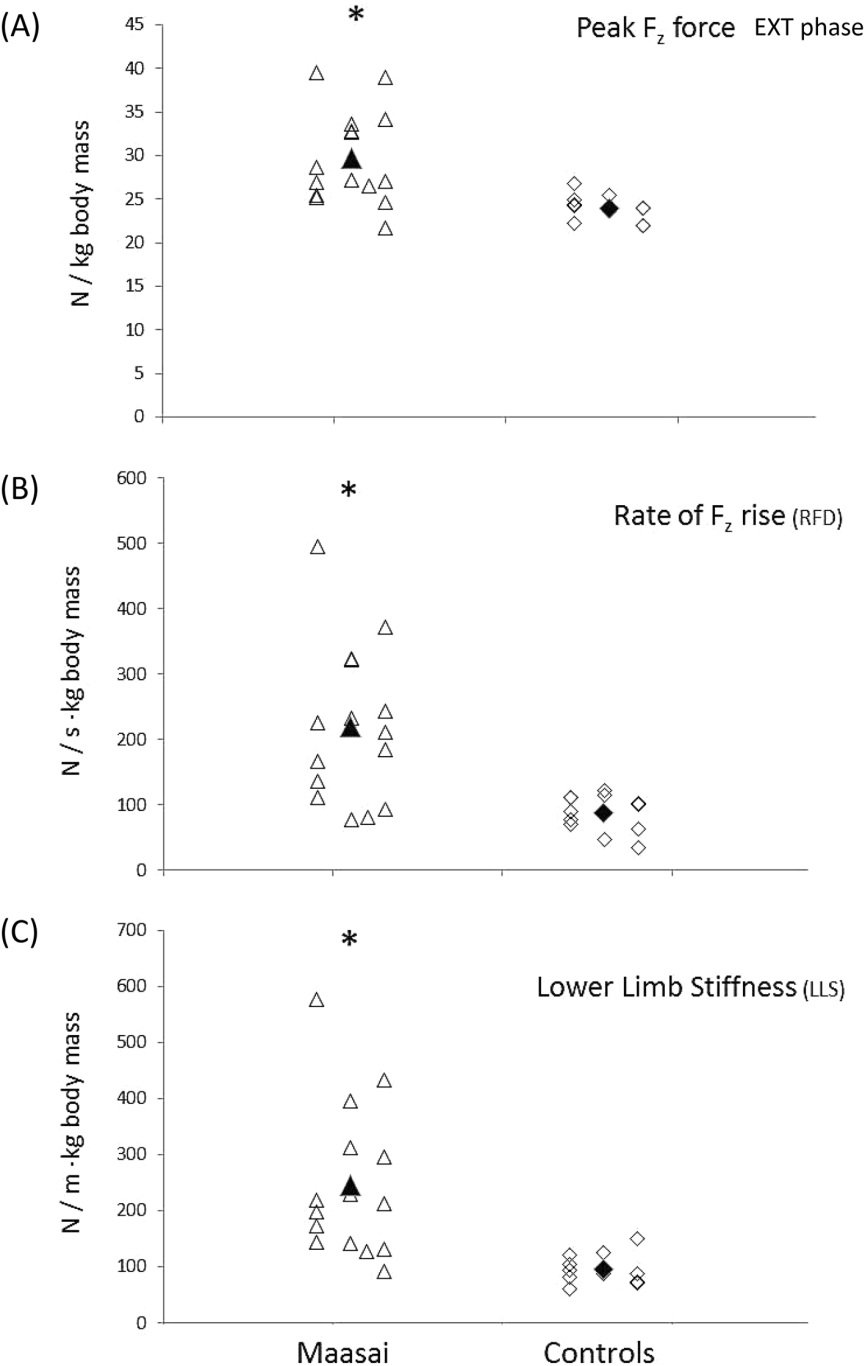
Countermovement jumping performance in Maasai (triangles) and CON (diamonds). Mean data fo the groups are depicted by solid symbols. (**A**) Peak vertical ground reaction force recorded in the extension phase of the take-off, (**B**) rate of force (F_z_) development (RFD), (**C**) lower limb stiffness (LLS). * Maasai vs. CON (*P* < 0.01).

**Fig. 5:**
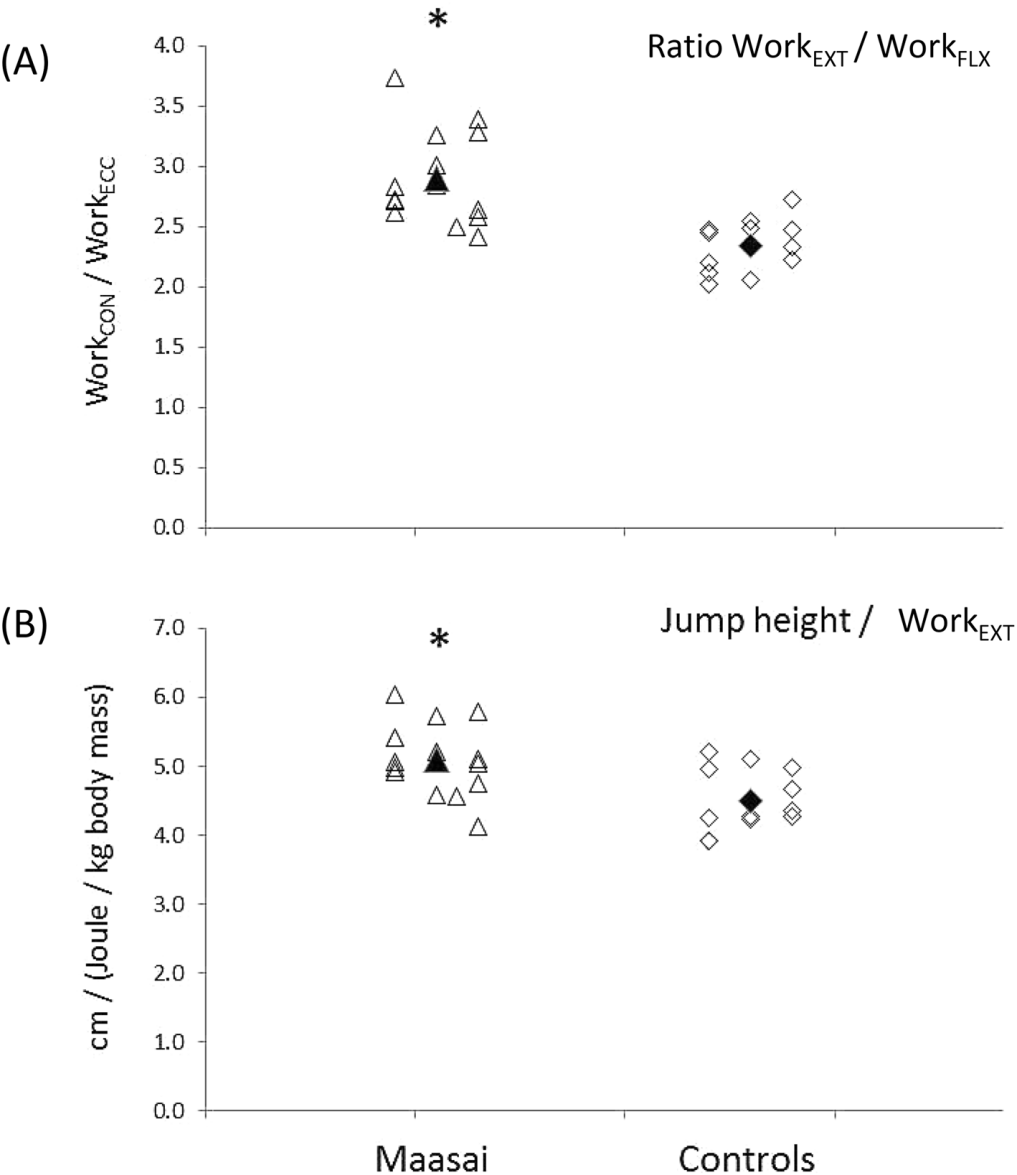
Countermovement jumping performance in Maasai (triangles) and CON (diamonds). Mean data of the groups are depicted by solid symbols. (**A**) Ratio of work performed in the extension phase to that of the flexion phase, (**B**) maximal vertical jump height normalized to work production in the extension phase as an indicator of jumping efficiency. * Maasai vs. CON (*P* < 0.01).

**Fig. 6:**
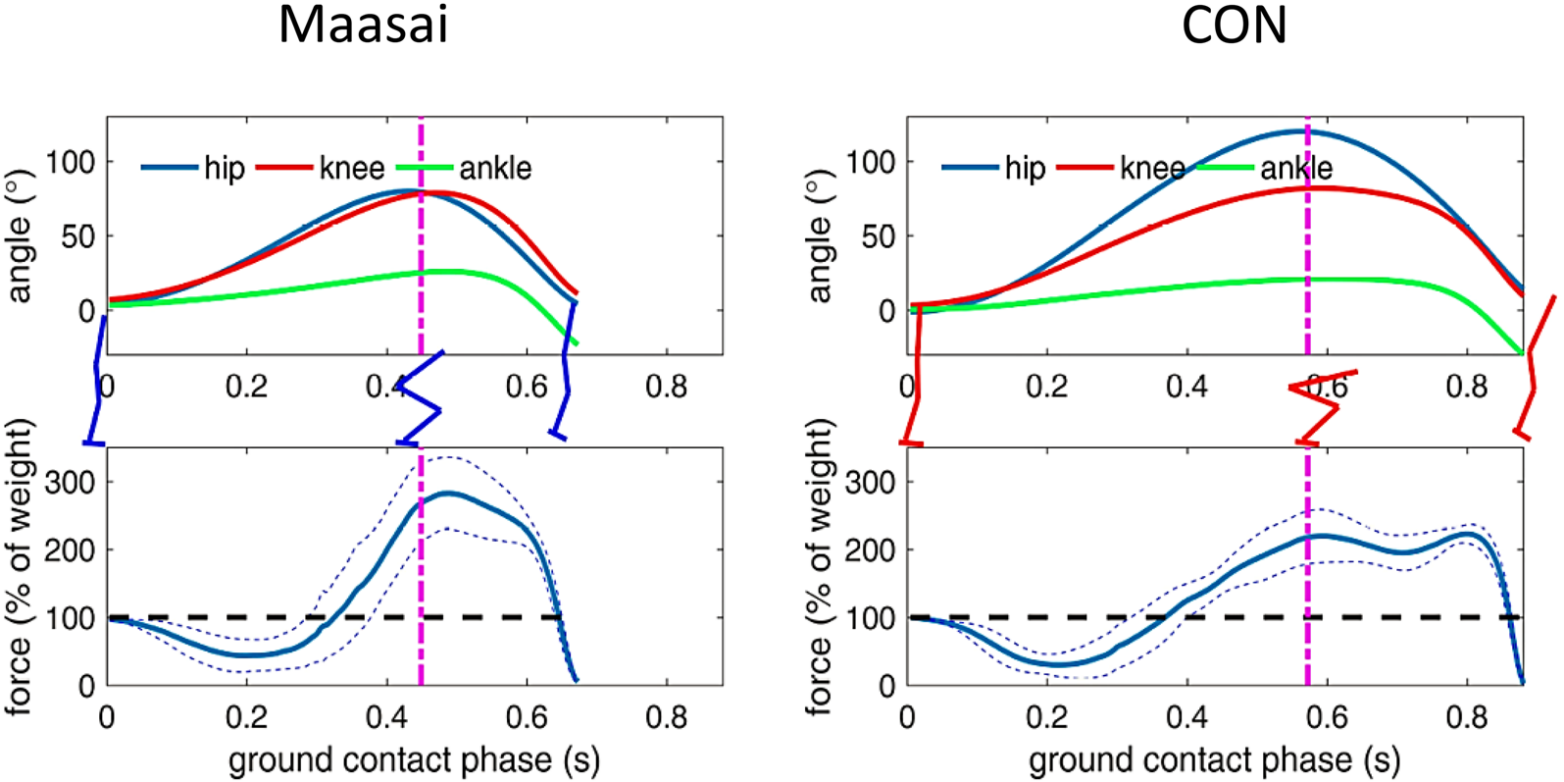
Kinematics during maximal CMJ: Upper panels; hip, knee, and ankle joint kinematics for the Maasai and CON respectively. Lower panels; Ground reaction force during the CMJ push-off phase.

In terms of jumping energetics, the Maasai demonstrated ~ 12% and 18% higher peak and mean BCM power during the propulsive extension phase (Table 3, Fig. 3A), explained by a 17% greater F_z_ force at P_peak_ in Maasai (*P* < 0.01) whereas vertical BCM velocity at P_peak_ did not differ between the Maasai and CON (Fig. 3B,C). Further, the Maasai participants showed 148% steeper RFD during the flexion phase (*P* < 0.01) along with 156% greater lower-limb stiffness (*P* < 0.001) (Fig. 4A,B,C), and 13% elevated vertical jump efficiency (*P* < 0.01) (Fig. 5B). Finally, the Maasai participants demonstrated a 23% elevated ratio of work performed on the BCM (extension to flexion phases) and a 43% greater RSI compared to CON (*P* < 0.001) (Fig. 5A). In terms of joint kinematics, the Maasai showed less joint range of motion (ROM) (− 24%, *P* = 0.001) at the hip joint compared to CON, while no group differences were seen for the knee and ankle joint ROM (Table 3 and Fig. 6).

### RJ

**Fig. 7:**
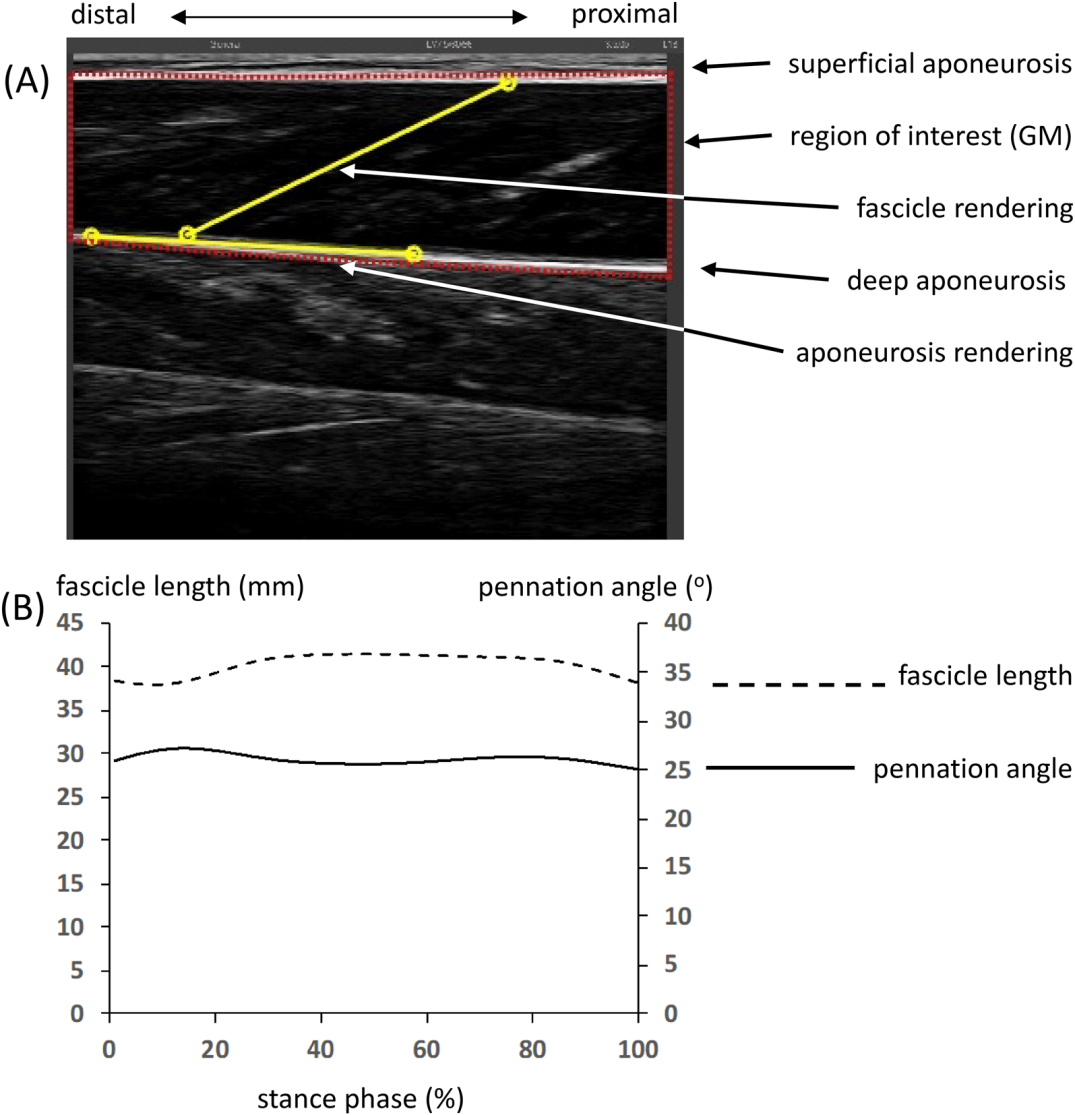
Muscle fascicle behavior during repetitive jumping. (**A**) Ultrasound image showing GM fascicle and aponeurosis tracking of one participant. The red area marks the region of interest. Yellow lines mark the fascicle and lower aponeurosis tracking. (**B**) Representative fascicle length (dashed line) and pennation angle (solid line) of the contact phase (0–100%) during maximal RJ of one participant.

Vertical jump height was not significantly different between Maasai and CON in both MRJ & SMRJ, although relative to body mass the Maasai participants jumped ~ 20% higher in both conditions (*P* < 0.001) (Table 4). In both jump types, the Maasai used a lower self-selected jump-frequency (MRJ ~ 10%: SMRJ ~ 15%, *P* = 0.015 and 0.029 respectively) accompanied by longer contact times (35% for MRJ and 39% for SMRJ, *P* = 0.011 and 0.001 respectively). Moreover, the Maasai participants showed greater hip and knee joint ROM during MRJ (hip joint: ~ 35%, knee joint: ~ 31%; *P* = 0.02 and 0.004 respectively) along with a greater ROM for the ankle joint in SMRJ (hip: ~ 78%, knee: ~ 52%, ankle: ~ 55%; *P* = 0.004, 0.001, 0.03, respectively) (Table 4, note that contrary to CMJ, data are not given as ROM, but instead as the maximally flexed/dorsiflexed joint angles during stance since the joint positions at initial landing was not strictly equal to *in situ* position). In terms of dynamic modulations in muscle architecture, muscle fascicle behavior was generally isometric (fascicle length changes in the contact phase ~ 1.5 mm corresponding to 3% of resting length) with no between-group differences (Table 5, Fig. 7). In all participants, the MTU demonstrated a classical stretch–shortening behavior during ground contact in both jumping conditions. Maximal elongation of the MTU was found to occur midway (50 ± 1%) in the ground contact phase. In both jumping conditions, maximal elongation of the MTU and tendinous tissue (TT) (normalized to standing MTU and TT length, respectively) was greater in Maasai than CON (4 & 7%. *P* < 0.01) (Table 5).

**Table 4:** Repetitive jumping I.

	Maasai (n = 9)	Controls (n = 8)	95% C.I	*P*
Maximal repetitive jumping				
Jump height (cm)	30.4 ± 6.2	29.7 ± 4.6	− 3.7, 5.1	0.795
Jump height/BW (cm/kg)	0.54 ± 0.11	0.44 ± 0.07	0.0, 0.2	0.044*
Contact time (ms)	302 ± 47	223 ± 63	22, 136	0.011*
Jumping frequency (Hz)	1.26 ± 0.05	1.41 ± 0.13	− 0.2, − 0.1	0.015*
Submaximal repetitive jumping				
Jump height (cm)	13.9 ± 3.9	13.9 ± 5.2	− 4.7, 4.7	0.988
Jump height/BW (cm/kg)	0.25 ± 0.01	0.20 ± 0.02	0.03, 0.07	0.216
Contact time (ms)	288 ± 24	207 ± 38	49, 113	0.001*
Jumping frequency (Hz)	1.61 ± 0.11	1.91 ± 0.35	− 0.6, − 0.1	0.029*
Kinematics. maximal repetitive jumping				
Maximal hip joint angle (°)	127.7 ± 12.6	141.8 ± 10.2	− 26.1, − 2.2	0.024*
Maximal knee joint angle (°)	109.7 ± 7.3	126.4 ± 12.8	− 27.3, − 6.1	0.004*
Maximal ankle joint angle (°)	25.0 ± 3.8 DF	21.7 ± 5.9 DF	− 1.8, 8.4	0.178
Kinematics. submaximal repetitive jumping				
Maximal hip joint angle (°)	146.1 ± 8.8	161.0 ± 9.3	− 24.4, − 5.5	0.004*
Maximal knee joint angle (°)	121.0 ± 7.3	141.2 ± 10.9	− 29.7, − 10.8	0.001*
Maximal ankle joint angle (°)	21.8 ± 5.2 DF	14.0 ± 8.1 DF	0.9, 14.8	0.030*

Group mean ± SD. 95% confidence interval (C.I.) for differences between means.

Data presented for both maximal and submaximal repetitive jumping at self-selected frequency. Data presented as an average of a number of cycles. Joint angles are presented as maximal flexion (hip and knee joint) and maximal dorsiflexion (ankle joint) during the landing relative to in situ joint position (hip joint and knee joint 180°; ankle joint 0°). DF denotes dorsiflexion.

*Denotes significant group difference.

**Table 5:** Repetitive jumping II: data presented for both maximal and submaximal repetitive jumping at self-selected frequency.

	Maasai (n = 9)	Controls (n = 8)	95% C.I.	*P*
Muscle–tendon mechanics. Maximal repetitive jumping				
Avg fascicle length, (range) (mm)	34.9 (34.0 − 35.4)	33.2 (31.0 − 34.4)	− 4.1, 7.5	0.523
Avg fascicle length/resting length	0.73 ± 0.10	0.70 ± 0.65	− 0.4, 0.5	0.240
Avg p. angle, (range) (°)	28.0 (27.2 − 28.7)	28.9 (27.5 − 30.89)	− 1.5, 0.1	0.722
Avg p. angle/resting p. angle (°)	1.4 ± 0.2	1.4 ± 0.3	− 0.2, 0.2	0.982
Max MTU length/sMTU length	1.17 ± 0.02	1.13 ± 0.02	0.0, 0.1	0.007*
Max TT length/sTT length	1.09 ± 0.02	1.03 ± 0.04	0.0, 0.1	0.002*
Muscle–tendon mechanics. Submaximal repetitive jumping				
Avg fascicle length, (range) (mm)	33.5 (32.8–34.0)	34.0 (32.9–34.5)	− 3.9, 4.6	0.865
Avg fascicle length/resting length	0.70 ± 0.11	0.67 ± 0.14	− 0.1, 0.2	0.635
Avg p. angle, (range) (°)	29.3 (28.6–30.1)	27.0 (26.4–27.6)	− 2.8, 3.1	0.367
Avg p. angle/resting p. angle (°)	1.5 ± 0.3	1.3 ± 0.1	− 0.1, 0.4	0.133
Max MTU length/sMTU length	1.15 ± 0.02	1.11 ± 0.02	0.02, 0.06	0.005*
Max TT length/sTT length	1.08 ± 0.02	1.01 ± 0.04	0.04, 0.1	0.001*

Fascicle length and pennation angles are presented as average (mm and °)during the stance phase of jumping, as range (mm and °), and relative to standing length (sMTU) and resting angle respectively. Group mean ± SD. 95% confidence interval (C.I.) for differences between means.

**Fig. 8:**
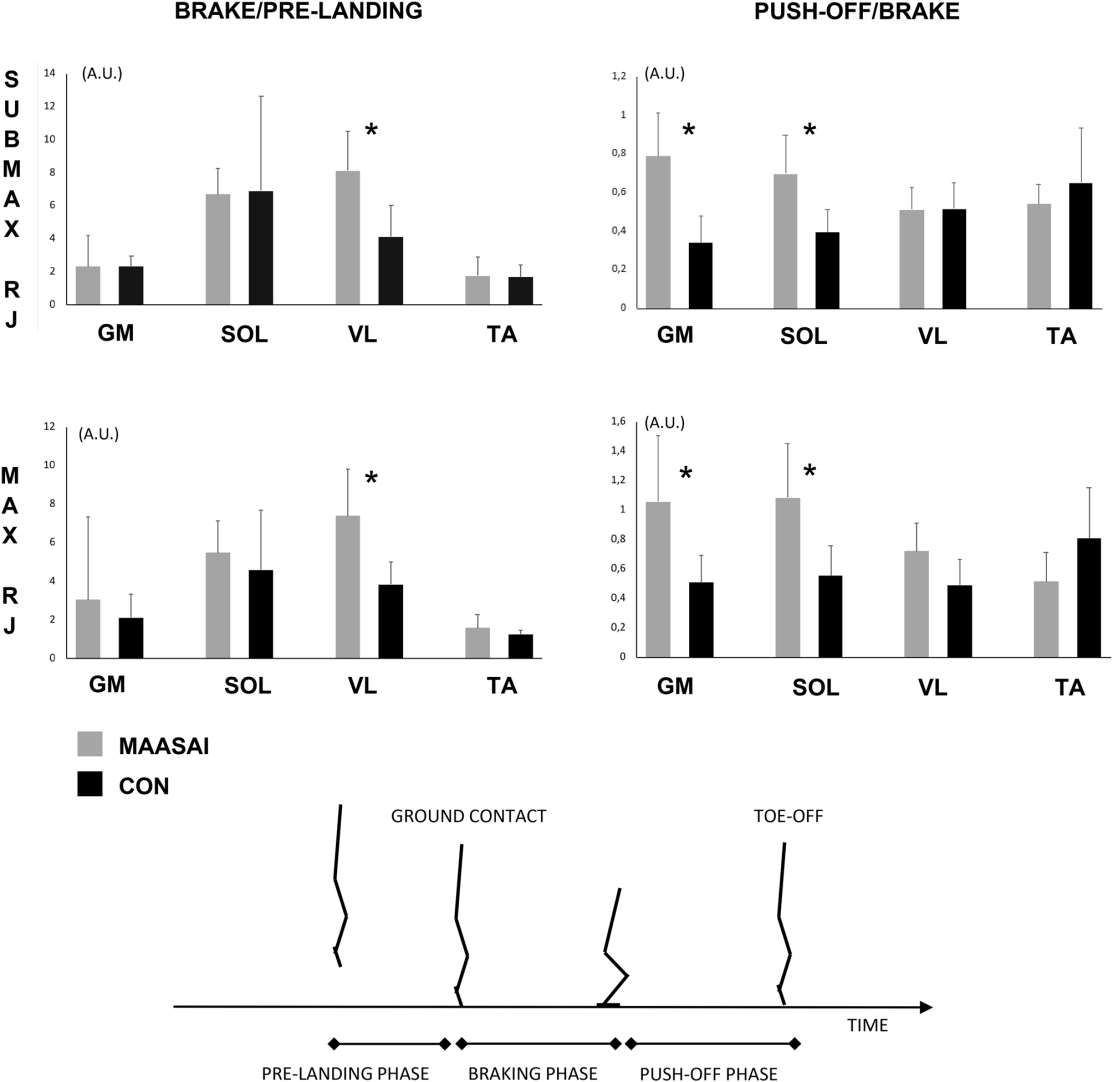
EMG ratios obtained during repetitive jumping (RJ). Ratio of averaged EMG (arbitrary units A.U.) in the braking phase relative to the pre-landing phase (left panels) and push-off phase relative to the braking phase (right panels). Submaximal RJ are depicted in upper panels, while maximal RJ is depicted in bottom panels. The pre-landing phase was defined as the 100 ms preceding first ground contact, while the braking phase was from ground contact to the instant of peak length of the GM-MTU (≈deepest point of BCM) during the stance phase, while the push-off phase was from the deepest point of BCM during the stance phase until toe-off. (GM; medial gastrocnemius, SOL; soleus, VL; vastus lateralis, TA; tibialis anterior). * Maasai vs. CON (*P* < 0.01).

Overall, EMG activity was greater during the braking phase compared to the pre-landing phase with the highest ratios observed in SOL and VL. In both RJ jump-types the VL ratio of braking to pre-landing was greater in Maasai than CON (SMRJ: 8 ± 2 vs. 4 ± 2, *P* = 0.006, (2.5, 5.59), MRJ 7 ± 2 vs. 4 ± 2, *p* = 0.043, (1.5, 4.5)) but no difference was seen between groups for other muscles (Fig. 8). Greater EMG amplitudes were observed during the braking phase compared to the push-off phase in both conditions, and the ratio of push-off to braking were significantly greater in Maasai for SOL and GM in both jumping conditions (GM: SMRJ 0.8 ± 0.2 vs. 0.3 ± 0.1, *P* = 0.007 (0.4, 0.6) and MRJ 1.0 ± 0.5 vs. 0.5 ± 0.2, *P* = 0.027 (0.2, 0.8) and SOL: SMRJ 0.7 ± 0.2 vs. 0.4 ± 0.1, *P* = 0.005 (0.2, 0.4) and MRJ 1.0 ± 0.4 vs. 0.6 ± 0.2, *P* = 0.012 (0.2, 0.7)) (Fig. 8).

## Discussion

The main findings in the present study were that the Maasai performed maximal CMJ in a very fast manner characterized by high take-off power, which illustrates a highly explosive jump execution in this group. Nonetheless, no group differences were observed in maximal vertical jump height during CMJ. In contrast, the Maasai showed a slower jump execution in RJ, with a greater joint ROM, and with a lower jump frequency compared to CON. Substantial anthropometric differences were seen with longer relative leg and shank length, a more proximal shank volume distribution, and a shorter plantar flexor moment arm of the Maasai. Further, the Maasai had shorter muscle fascicles, lower pennation angles, and lower muscle thickness, whereas the triceps surae tendinous structures were longer and more compliant compared to CON.

### CMJ performance

Although some Maasai participants showed outstanding individual jump performance (Maximal jump height: Maasai: 46.3 cm vs. CON: 43.1 cm), no systematic group difference could be detected in maximal vertical jump height, which seems contrary to anecdotal reports. Yet there were distinct differences in the execution of the maximal jumping tasks: The Maasai performed very brief push-off actions with shorter phase times (flexion and extension phases) and elevated leg extensor power production. In terms of kinematics, the maximal knee and ankle joint angles during push-off were similar between groups, but the Maasai had a markedly smaller hip joint flexion relative to the in situ position, and thus the magnitude of vertical BCM displacement during the push-off phase was significantly less in the Maasai. For jumping kinetics, the Maasai produced significantly greater peak and mean ground reaction force and power (in both flexion and extension phases), and notably the dynamic RFD and the apparent LLS was ~ 150% greater in the Maasai. RFD and LLS data obtained in the CON group were comparable to those previously reported in untrained young male adults^20^, but we have no knowledge of previous reports that report RFD of magnitudes seen in the Maasai. With respect to power exertion normalized to body mass, the present group of Maasai showed similar magnitudes of P_peak_ during take-off (55 W/kg) as previously reported with CMJ testing in power lifters (57 W/kg), whereas Olympic lifters and US National level sprinters produced significantly higher P_peak_ values (63 W/kg). In these previous reports^20,21^ untrained individuals were found to produce markedly less P_peak_ (49 W/kg), which, corresponds to present values seen in CON. Overall, for peak and mean power produced during the extension phase, the Maasai participants presented values comparable to, or exceeding previously reported values for elite level explosive-type jumpers^21,22^, and very high jumping efficiency compared to CON when expressed as jump height/work produced in the extension phase and reactive strength index (RSI).

In general, the MTU can be considered a specialized propulsion actuator where the in-series design of contractile and passive tissue functions as a power amplifier by a time-wise decoupling of the contractile work from that of the locomotor task, which yields greater overall power^14^. It thus seems reasonable to speculate, that other than differences in neural activation or muscle physiology (e.g. fiber type differences), the observed differences in maximal jump execution between the groups may at least in part relate to the differences in muscle–tendon morphology that was observed between Maasai and CON.

### RJ

Maximal RJ height was similar between groups, but also in this task, the execution of the jumps differed: The Maasai jumped with a lower frequency and longer ground contact times compared to CON, which contrasts to the CMJ where the Maasai executed the jump markedly faster than CON. Compared to maximal CMJ, RJ is a vastly different movement task where efficiency likely plays a more important role. Thus, in RJ the MTU may act as an energy conservator where tendon length changes likely contributes to storage and release of mechanical energy, and thus by modulation of neural activation and muscle fiber contraction properties, the movement efficiency is augmented in such a movement task^16^. CMJ is a maximal stretch–shortening cycle movement, and it has been discussed previously if energy storage and release is important for performance (maximal jump height), or if the main role of the force transmitting tissues lies in optimizing (potentiating) the active state of the contractile apparatus to hereby maximize force production and peak power output^23,24^. While the pattern of shortening/elongation of the contractile tissue and the tendinous tissues during RJ in the current study was similar to what has previously been reported using similar methods (one legged hopping^16^), the lower RJ jumping frequency observed in the Maasai also contrasts with the notion of the leg resonance frequency being inversely proportional to body mass^25^. Based on this notion, the leg resonance frequency of the Maasai participants would be estimated to be greater than in CON, which in turn would be expected to favor a higher frequency in the Maasai compared to CON. Thus, the reason why the Maasai adopt a lower RJ frequency remains unclear, although speculatively it could be related to the observed more compliant tendon structures. The architecture of the Maasai MTU was characterized by relatively longer tendons and shorter muscles, which are typical features associated with economical force production^26^. Whether these anatomical differences relate to the reduced RJ frequency is not known, and it may be that RJ patterns of the Maasai simply reflect a favored jumping style related to other aspects than efficiency.

Although the present EMG data should be interpreted with caution due to the low number of participants involved in this analysis, it seemed in both RJ types, that the relative activation of the knee extensor muscles was greater relative to the pre-landing phase in the Maasai. Combined with the present observation of greater knee joint ROM in the Maasai, these data may correspond with previous reports of amplified knee extensor muscle activation and enlarged knee joint excursion to modulate jumping amplitude in RJ^27^. The Maasai displayed a greater push-off to braking phase EMG ratio for the plantarflexor muscles compared to CON. In contrast, Kenyan world-class runners showed a lower EMG push-off to braking phase ratio for the plantarflexor muscles during RJ testing^17^, using similar methods. Although difficult to interpret, it seems that the Maasai utilize a different jumping strategy in RJ, which may relate to their morphological features, or a training effect associated to frequent RJ activities, or simply result from a specific low-frequent cultural jumping pattern that by tradition involves low frequency RJ different from maximal fast RJ.

### Anthropometry

In the present study, relative leg length and shank length of the Maasai were greater than those of the CON. While no previous reports exist for Maasai, Kenyan runners who are mainly of Kalenjin origin have comparable anthropometric features i.e. demonstrating long legs relative to body height^28,29^. While absolute foot length was similar in the two groups, relative foot length was greater in the Maasai. The absolute and relative (% shank length) Achilles tendon moment arm was significantly shorter in the Maasai compared to CON, but similar to what previously was reported in Kenyan runners^29^. Maasai men are anecdotally known for a considerable stature, but previous scientific studies have documented a height of approximately 165–174 cm, a body mass of 59–64 kg, and a BMI of 19–21 kg/m^2^^2,11,12^, and the height of the present Maasai participants was thus similar to that of previous reports^2,11^, while current body mass and BMI seemed slightly lower^2,11,30^.

### Muscle architecture

No differences between groups were seen in muscle thickness, pennation angle or fascicle length for the thigh, but for the calf muscles fascicles were shorter, pennation angles shallower and muscle thickness less in the Maasai compared to CON. The surprising discrepancy between thigh and calf muscles suggest that any between-group difference in performance or jump execution may at least in part relate to differences in plantarflexor muscle-architecture. In a recent study, the reported MG fascicle length (48.0 ± 7.0 mm) and pennation angle (20.6 ± 2.2°) in Kenyan runners^29^, appeared similar to those seen in the present Maasai participants, which suggests a high degree of muscle-architectural similarity between these populations.

One previous study examined muscle architecture, Achilles tendon moment arms and foot length in sprinters, and found longer plantarflexor fascicles and shorter moment arms in sprinters compared to non-sprinters and the consequently improved gear-ratio was suggested to contribute to improved generation of explosive forward propulsion impulse^31^. Despite the shorter Achilles tendon moment arms of the present Maasai participants, the group did not demonstrate longer fascicles or larger fascicle/moment arm ratios, and thus did not seem to exhibit a plantarflexor apparatus comparable to that reported for sprinters. Consequently, the current observation of explosive-type CMJ execution in the Maasai is likely to relate to other physiological (neural activation and/or muscle fiber type composition) or biomechanical variables. Nonetheless, although previous observations underscore how anatomical features may influence movement performance, the rather distinct anatomical differences between groups may have influenced contractile dynamics but did not functionally translate into difference in absolute maximal jump height between groups in the present study.

Muscle volume was not directly assessed in the current study, but the calf circumference combined with the lower calf muscle thickness of the Maasai indirectly suggests lower plantar flexor muscle volume compared to CON. Moreover, the Maasai appear to have a more proximal distribution (shorter relative calf muscle length) of plantarflexor muscles, which does not necessarily relate to e.g. maximal jumping performance, but it is known to contribute to overall efficiency during human movement^32^. A previous study^11^ reported leg circumference measurements in Maasai with data like the present numbers. The present and previous data on shank muscle volume in the Maasai thus seem in accordance with prior notes of leg circumference of Kenyan boys that were compared to Danes of similar age^28^.

### Achilles tendon properties

Previous studies have suggested that the elongation of the series elastic element during maximal propulsion efforts such as jumping may modulate the ability to exert muscle force and power by a timewise decoupling of muscle work and external work^14,23,33^. The free Achilles tendon length was similar between groups, but the GM tendon length, i.e. the length of the entire force transmitting structure for the GM, was markedly longer and more compliant in the Maasai compared to CON. Thus, the series elastic element of the GM showed a markedly greater potential for lengthening for the Maasai, which theoretically could affect jumping performance positively. The role for Achilles tendon stiffness in maximal jump performance is not entirely clear, but previous studies^19,34^ have indicated a positive relation between tendon stiffness and isometric RFD, which is considered an important feature in explosive type movements^35^. In support of this notion, positive correlations have been reported between tendon stiffness and maximal vertical jumping performance^19^ although these data were for the tendon structures of the knee extensors. It should be noted that maximal vertical jumping is a complex task involving the entire leg extensor apparatus, and that while patella tendon stiffness may contribute positively to maximal jump performance, it could be opposite for the Achilles tendon^36^ where added compliance can potentially enable greater energy storage/release and thus contribute to power output of the system. Nonetheless, the difference in tendon stiffness between groups did not seem to translate into differences in maximal jumping height between groups.

In the present study, the ankle joint angle in the resting prone position was greater for CON such that the unloaded ankle joint assumed a more plantarflexed position. The significance of this observation is not clear, but resting ankle angle may reflect the balance in passive tension of agonist–antagonist MTUs that in turn may have functional consequences for active performance about the ankle joint. It may seem that the more dorsiflexed position of the Maasai could reflect the more compliant force transmitting structures of the plantarflexor MTU.

### Economy of movement

Studies of human locomotion^37^ suggest that the work required for lower limb movement represents a substantial portion of the overall metabolic cost, which has been shown by adding mass to the leg/foot during running^38,39^. Based on such data, it was hypothesized that the superior running economy of Kenyan runners may relate to the anatomy of the lower limbs with a proximal mass distribution^28,40^, and it could thus be speculated that some of the presently observed anthropometric features of the Maasai would also contribute to economy of movement during locomotion. A shorter absolute and relative Achilles tendon moment arm was seen for the Maasai compared to CON, which was similar to that of Kenyan elite runners^29^. A shorter moment arm has been associated with better running economy^41^, and experimental and modelling data indicate that a shorter moment arm and a greater tendon length markedly increases gross efficiency during repetitive hopping^42^. Likewise, short fascicles (and long leg segment lengths) are known to reduce the cost of movement (in different animal species)^43,44^, and recent human modelling data suggest that an optimal combination of shorter fascicle lengths, low muscle volume and compliant tendon yields greater economy of movement during walking^32^. The present investigation did not assess economy of movement, however, the considerations above may suggest that Maasai men may express superior economy of movement during walking and running, and perhaps during repetitive jumping as well.

### Limitations

The present cross-sectional data were acquired in a field setting with numerous practical, technical, and logistical challenges. Specifically, despite familiarization procedures, the Maasai participants were unaccustomed to CMJ testing, while in turn the Norwegian control group was not familiar to RJ. This could render comparison between the groups somewhat difficult with respect to jump execution/performance. The two groups of participants was matched for age, and although it was attempted to also match the groups for body mass, this was unattainable. Further, CON participants were significantly taller compared to the Maasai, which also limits the interpretation of the present between-group comparisons. In addition, the small number of study participants could compromise the applicability of the data within both populations. However, time constraints and factors related to the experimental setting strongly limited the availability of participants. Importantly, recent changes in life conditions of the Maasai, which at the overall level is characterized by the encroaching urbanization of rural areas, may have had an impact on weekly exposure to jumping activities. Finally, we included Maasai with an age range between 18 and 37 years, thus spanning from individuals of *Moran* status to individuals who have been through the coming-of-age ceremony called *Eunoto*, allowing Maasai men to marry^8^ which likely implied heterogeneity with respect to habitual participation in jumping-dance events.

### Conclusion

Although some individual Maasai men demonstrated outstanding maximal vertical jump performance no systematic between-group differences in maximal absolute jump height were observed. However, maximal CMJ execution differed substantially between groups as the Maasai demonstrated a much faster take-off phase along with greater ground reaction forces, higher power generation, elevated limb stiffness and superior vertical jump efficiency compared to CON. For a number of these variables, the Maasai demonstrated values comparable to those previously reported in highly strength trained athletes. In contrast, RJ was performed with a much lower jump frequency by the Maasai albeit with similar jump height compared to CON. Thus, CMJ and RJ jump execution differed markedly between groups, which may relate to group experience with and tradition for jumping but could also rely at least in part on differences in anthropometry and biomechanical MTU properties, including muscle architecture and mechanical properties of the series elastic element. From previous studies it seems that the anthropometric and muscle–tendon morphological/mechanical properties observed in the present Maasai participants point towards greater economy of movement during walking. Consequently, future studies should explore whole body metabolism and the economy of movement during different horizontal gaits and perhaps during RJ as well.

## Materials and methods

The current study is part of a larger investigation that has examined cardio-metabolic health and physical activity, in Tanzanian and Kenyan Maasai people^8,30^. Here, we report biomechanical data from a field study carried out in Monduli district (Arusha Region) of Tanzania. Physical activity and metabolic data during jumping-dancing activity in the same Maasai participants are presented elsewhere^8^.

Through a local Maasai co-investigator (JS), based on availability, 22 Maasai men from villages in the rural area of the district capitol of Monduli Town, volunteered to participate in the study. Self-reported habitual weekly jumping dance frequency for the Maasai was 3.8 ± 1.9, with a duration per session of 2.4 ± 2.1 h. Twelve Norwegian men (white European origin, recruited from the Oslo area, Norway) volunteered to participate as a control group (CON), and CON tests were carried out at the Norwegian School of Sports Science (Oslo, Norway). The CON participants were recreational athletes with no specific jump-training history, but participated weekly in activities like recreational running, ball games and strength training. We attempted to match the two groups for body weight, but this was not possible given the disparate anthropometric population characteristics. Inclusion criteria for all participants were male, 18–40 years of age without any known or recent history of serious injury/illness, and with the ability to perform the protocol. CON was asked to refrain from exercise or high intensity training 24 h prior to testing, while similar requirements were not possible to apply for the Maasai participants.

During all measurements in Monduli district, an English and Maasai speaking interpreter was present to assist in explaining the procedures. The study was conducted in accordance with the Declaration of Helsinki and approved by the local Ethics Committee in Tanzania (Tumaini University, Moshi, certificate no. 507). Each participant signed an informed consent form and details were explained carefully to all participants prior to participation.

### Overall experimental design

Each participant underwent one laboratory session during which anthropometrics was recorded and resting muscle thickness, fascicle length and pennation angle of leg muscles (gastrocnemius medialis (GM) and vastus lateralis (VL)) were measured by use of ultrasonography. Achilles tendon stiffness was determined from synchronized recordings of tendon force and elongation. Maximal countermovement jumps (CMJ) were performed, and finally, to reflect the ritual jumping movement of the Maasai, bouts of both maximal and submaximal repetitive jumping (RJ) were carried out. All jumping was performed on a force plate to record ground reaction forces, while also kinematic, ultrasonographic, and electromyographic (EMG) data were obtained.

### Anthropometry

Body height was measured with a portable stadiometer (Meterex II, D97, UNICEF, Copenhagen, Denmark), while body mass was assessed with a high precision scale (BWB-800 SMA, Tanita, Tokyo, Japan). Leg length was defined as the distance from the greater trochanter of the femur to the floor while standing barefoot. Thigh length was measured from the greater trochanter to the lateral knee joint line, while shank length was recorded as the distance from the knee joint line to the floor. Volume distribution of the shank was estimated by measuring leg circumference (standing) at nine different sites (10% increments from proximal to distal) of the shank.

With participants lying relaxed in the prone position, and with feet hanging freely over the edge of the bench, sagittal-plane photos were taken laterally to determine the resting ankle joint angle defined as the angle between the lower leg and the lateral aspect of the foot sole, and presented in ° plantar/dorsiflexion (PF/DF) relative to neutral (0°) . The Achilles tendon insertion on the calcaneus, the most distal insertion of soleus (SOL) on the Achilles tendon, and the most distal insertion of the GM on the aponeurosis were identified by ultrasonography (LogicScan 128 EXT-1Z, linear 60 mm transducer, scanning frequency: 12 MHz, HL9.0/60/128Z-2, Telemed, Lithuania) and marked on the skin. The distance from the insertion of the Achilles tendon to the point of the most distal insertion of SOL was noted as the free Achilles tendon length, while the distance from calcaneal insertion to the most distal GM insertion on the aponeurosis was defined as the the GM tendon length^45^. The distance from the most distal GM insertion to the most distal SOL insertion was defined as the length of the GM aponeurosis^45^. Achilles tendon moment arm and foot length were measured in standing position from sagittal plane photos^29,41^, using imaging software (Fiji, ImageJ^46^).

### Resting muscle architecture

Longitudinal ultrasound images of the GM muscle were acquired at the 30% mark of the GM muscle length (see above), and for the VL at 40% of the length from the knee joint line to the greater trochanter. From these scans, the muscle fascicle pennation angle and fascicle length were obtained by use of imaging software (Fiji, ImageJ^46^) as described previously^47,48^. From transversal scans acquired at the same sites, muscle thickness was determined as the shortest distance between the superficial and profound aponeuroses.

### Achilles tendon stiffness

The tensile stiffness of the Achilles tendon structure (GM tendon) was examined by synchronous recordings of ultrasound videos (> 38 Hz) of tissue displacement and plantar flexor torque during isometric ramp contractions^45,49–51^. The ultrasound probe was placed sagittally over the distal muscle–tendon junction of GM and fixed to the leg using a custom-made rigid cast to enable stable positioning with minimal tissue compression. The participants were seated in a rigid custom-made portable isometric dynamometer instrumented with a load cell (U2A 500 Hottinger Baldwin Messtechnik, Darmstadt, Germany). The dynamometer had firm lower back support, and participants were seated with appr. 90° hip flexion, the knee straight, and the foot at neutral ankle joint position. Undesired joint angle movements were minimized by careful strapping of the limbs and further ensured by the rigid design of the dynamometer that included firm support of the pelvis/lower back.

Following careful explanation and familiarization to the procedures, three isometric (10-s) plantar flexor ramp contractions from zero force to maximal voluntary force were carried out, separated by 2 min of rest. Prior to the ramps, five brief sub-maximal contractions served to pre-condition the tissue^52^. The force signal and video quality were evaluated, and additional trials were carried out if necessary. Tendon force was derived from load cell data and moment arm calculations^45,51,53^. Displacement of the GM muscle–tendon junction was measured using a semi-automated tracking algorithm (Tracker 4.11.0, Open Source Physics, Aptos, California, USA)^48^, to represent tendon elongation. For each individual, valid trials were averaged. The individual tendon force and elongation data were truncated at 90% peak force and subsequently fitted with a second-order polynomial (R^2^ = 0.96–0.99). The tendon stiffness was defined as the slope of the fitted curve between 60 and 100% force^51^.

### CMJ

Following instruction and familiarization jumps, participants performed 3–5 maximal CMJ on an instrumented force plate (further detail given below) and the attempt with the highest vertical jump height was selected for further analysis^19,54^. Vertical ground reaction force (F_z_) data were sampled at 300 Hz and subsequently low-pass filtered (Butterworth 4th-order zero-lag filter, cut-off frequency 100 Hz) and exported to the motion analysis software Qualisys Track Manager. Picture frames of the Oqus 400 cameras were synchronized to analog signals running into the USB analog board using a separate sync cable (details given below). The onset of the jump was defined as the time point where F_z_ went 5N below the mass (N) of the participant, while the instant of take-off was identified as the time point where F_z_ fell below 0.5% of F_z_ peak-to-peak amplitude (≈10N)^19,54^. BCM acceleration was calculated by dividing the F_z_ signal by body mass and subtracting gravitational acceleration (g = 9.81 m/s^2^). Successively, the acceleration signal was numerically integrated to yield BCM velocity, which in turn was integrated to yield BCM position. Finally, instantaneous BCM power was calculated continuously through the jumping movement as the product between F_z_ and BCM velocity^19,54–56^. Vertical jump height (vertical BCM displacement relative to the instant of take-off) was determined from the take-off velocity using previously reported calculations^19,54–57^. The duration of the ‘*flexion phase’* (BCM moving downwards), the deceleration during the flexion phase, and the propulsive ‘*extension phase’* (BCM moving upwards) during the CMJ take-off was determined, and peak F_z_ during flexion and extension phases were identified along with the mechanical work performed on BCM during both phases. Mean and peak BCM power (P_peak_) was determined in the extension phase, and dynamic rate of force development (RFD) was derived as the slope of the F_z_ curve calculated from onset of deceleration (instant in the flexion phase where F_z_ > body mass) to + 50 ms^20,54^, while lower limb stiffness (LLS) was derived from corresponding values of F_z_ and BCM position^20,57^. Reactive strength index (RSI) was calculated as jump height (m)/duration of the FLX_dec_ + EXT phases (s), and finally, mechanical jumping efficiency was calculated as maximal vertical jump height divided by the magnitude of work produced on the BCM during the extension phase.

### RJ

Two series (~ 30-s duration) of RJ were performed. In the first bout the participants jumped at a preferred (submaximal) pace and intensity (SMRJ). Participants were asked to jump at a comfortable, non-fatiguing pace, without any instruction regarding jump height. In the second bout, the participants were asked to perform maximal repetitive jumps (MRJ). All jumping was performed akimbo. Familiarization jump series were conducted prior to data sampling. The initial 10–15 s within each sweep served as additional habituation and once the jumping was deemed stable (stable jumping around the center of the force plate), the recording (~ 10-s) of ground reaction forces, EMG, kinematics and ultrasonography was initiated. RJ series were separated by ~ 2 min rest periods and attempts were repeated in case of sampling error or unstable jumping. For technical reasons (limitations related to shipping of equipment), two different force platforms were used: A lightweight force plate (Biomekanikk AS, Oslo, Norway) was used for the Maasai participants while an AMTI (BP6001200, AMTI, Watertown, MA, USA) force plate was used for CON participants.

During RJ, ultrasonography (LogicScan 128, 60 mm linear array probe (scanning frequency: 7 MHz, LV7.5/60/96Z), Telemed, Vilnius Lithuania) was applied to measure GM fascicle length and pennation angle. The ultrasound probe was secured tightly to the right leg using a custom-made cast to minimize probe movement. Depending on the dynamic range of the image, the frequency of the ultrasound recordings was between 72–84 images/s, with a 50 mm image depth. Ultrasound videos were saved in the uncompressed AVI-file format (Echo Wave II (3.4.2b5, Telemed, Lithuania) for subsequent analysis.

### Electromyography

EMG signals were obtained from the left leg during RJ using bipolar surface electrodes (Ag/AgCl, size: 45*22 mm, Neuroline 720 72,000-S, Ambu, Ballerup, Denmark) with an inter-electrode distance of 20 mm. Skin preparation and positioning of the electrodes adhered to SENIAM guidelines^58^ with a reference electrode placed on the medial tibial surface. EMG electrodes for MG, SOL, tibialis anterior and VL were connected to an individual Noraxon DTS EMG wireless transmitter (Noraxon Inc., Scottsdale, AZ, USA) and the EMG signals were transmitted to a stationary telemetric receiver (TeleMyo DTS Desk Reciever, Noraxon Inc., Scottsdale, AZ, USA). EMG was thus recorded from the contralateral leg (relative to the ultrasound probe) due to space constraints. EMG sampling frequency was set to 1500 Hz and the signal was hardware band-pass filtered (5–500 Hz), and during later offline analysis full-wave rectified and low-pass filtered with a Butterworth zero-lag 4th-order filter (10 Hz cutoff frequency)^59^.

Due to time and logistic constraints in the field setting, it was not possible to perform EMG recording during maximal voluntary contractions for reference. Thus, the interpretation of EMG signals during RJ was limited to patterns of relative EMG activity during the pre-landing, braking and push-off phases, respectively. Comparable to previously published methods on muscle activation during repetitive jumping^17^, the pre-landing phase was defined as the 100-ms preceding the instant of ground contact while the braking phase was defined from the time of ground contact to the time point of peak elongation of the GM muscle–tendon unit (MTU) during the stance phase. The push-off phase was defined from the time of peak GM MTU elongation to instant of take-off. A root mean square envelope (50 ms time constant) was applied to the EMG signal before individually averaging the signal for each phase. The ratios of averaged EMG RMS amplitudes between specific movement phases (braking phase to pre-landing and push-off to braking) were calculated^17,29^.

### Kinematics

To ensure synchronized recording of kinetic, kinematic, ultrasound and EMG signals, data collection was simultaneously triggered from the ultrasound scanner. Data were saved in Qualisys Track Manager (Qualisys, AB, Gothenburg, Sweden) for subsequent analysis. For RJ, contact time and jumping frequency were determined from the ground reaction force signal while jump height was determined from flight time^60^.

During CMJ and RJ, two-dimensional kinematic data were recorded with three retro-reflective cameras (Oqus 400, Qualisys, AB, Gothenburg, Sweden) at 300 Hz frame rate. Prior to jumping, six 12-mm reflective markers were placed on anatomical landmarks (acromion, the greater trochanter of the femur, the lateral epicondyle of the femur, the lateral malleolus of the tibia, the posterior aspect of the calcaneus, and on the fifth metatarsal head). During subsequent analysis, all position data were filtered using a Butterworth 4th-order low-pass filter with a cut-off frequency of 10 Hz. During the stance phase joint angles were determined as an average of three cycles obtained in each of the conditions.

### Muscle–tendon mechanics during RJ

Ultrasound videos were analyzed using a semi-automated tracking algorithm^61,62^ to obtain muscle fascicle lengths and fascicle pennation angle (Fig. 7). Hip, knee, and ankle joint angles were determined from kinematic recordings as described above and GM MTU length was calculated based on the equation reported by Hawkins and Hull^63^, while instantaneous GM tendon length was calculated using the equation reported by Sano & coworkers^17,29^.

#### Statistical analysis

For technical and practical reasons related to field study conditions (temporary equipment failure and low-quality imaging) the number of participants differed between the specific analyses, as specified in the results section. All data samples were checked for normality (Shapiro–Wilk test/Q–Q plot), and differences between Maasai and CON groups were analyzed using two-tailed Student’s independent samples t-test (for normally distributed samples), while the non-parametric Mann–Whitney U test was applied when a normal distribution could not be detected. Nonparametric testing was applied in 4 comparisons (CMJ-RFD, LLS, relative pennation angle in MRJ & SMRJ). The overall alpha level was set to *P* ≤ 0.05, and results are reported as group means ± standard deviation (SD) 95% confidence intervals C.I. for the difference between means are given in parentheses.

## Data Availability

The datasets generated during and/or analyzed during the current study are available from the corresponding author on reasonable request.
